# Human Endothelium-on-a-Chip: Development of a Microfluidic Model for Cell Viability Assessment Under Oxidative Injury

**DOI:** 10.3390/ijms27115018

**Published:** 2026-06-02

**Authors:** Klemen Kirbus, Jakob Kolar, Črt Krebs, Petra Kocbek, Lovro Žiberna

**Affiliations:** 1 Faculty of Pharmacy, University of Ljubljana, Aškerčeva cesta 7, 1000 Ljubljana, Slovenia; klemen.kirbus@ffa.uni-lj.si (K.K.); jakob.kolar@ffa.uni-lj.si (J.K.); petra.kocbek@ffa.uni-lj.si (P.K.); 2 Faculty of Medicine, University of Ljubljana, Korytkova ulica 2, 1000 Ljubljana, Slovenia; crt.krebs@mf.uni-lj.si

**Keywords:** endothelium, organ-on-a-chip, microfluidics, oxidative stress, shear stress, cell viability

## Abstract

Endothelial responses to oxidative stress are influenced by the presence of blood flow and shear stress. To better capture these conditions in vitro, we developed an endothelium-on-a-chip model for the study of acute oxidative injury in EA.hy926 cells under flow. We adapted the culture conditions for use outside a conventional 5% CO_2_ atmosphere, and we improved endothelial adhesion and retention under flow by collagen coating of the chip surface. Our model tolerated shear stress of up to 0.89 Pa, which corresponds to the in vivo values experienced by endothelial cells in the aorta and venous system. On this model, we designed an acute oxidative injury protocol based on high concentrations of hydrogen peroxide (H_2_O_2_) as an endogenous source of oxidative stress and 2,2′-azobis(2-amidinopropane) dihydrochloride (AAPH) as an exogenous molecule. The inclusion of flow increased the apparent sensitivity of the model to oxidative injury compared to static conditions, as the IC_50_ values decreased from 16.2 mM to 12.0 mM for H_2_O_2_ and from 100.7 mM to 72.5 mM for AAPH under flow conditions. Pretreatment with quercetin (1.5 µM) reduced AAPH-induced oxidative injury, thus showing the potential applicability of the model for antioxidant screening assays.

## 1. Introduction

The preclinical development of new active pharmaceutical ingredients has long been associated with large-scale animal studies. With the advent of novel cell-based technologies, animal use in preclinical studies can now be reduced, and is even no longer necessary for registration of novel drugs in the USA, according to the FDA Modernization Act 2.0 [1]. Moreover, novel cell-based technologies enable more cost-efficient pharmaceutical development and, in some cases, even better translational value from pre-clinical to clinical studies [2,3]. One such novel technology is organs-on-a-chip (OOC), which are distinguished from other cell culture models by the incorporation of culture media flow and establishment of complex intercellular communication in a 3D extracellular matrix [3].

The exposure of cells to flow of culture medium can significantly change cell behavior compared to static conditions. This is especially important for in vitro studies of the vasculature and microvasculature to mimic in vivo conditions where endothelial cells are continuously exposed to shear stress [4,5,6]. OOC systems have therefore been increasingly used to establish models of endothelial barrier (dys)function in disease [5,7], migration of neutrophils [8], inflammation [9,10], and atherosclerosis [11,12].

The vascular endothelium is crucial for maintaining multiple body functions, such as hemostasis, vascular tone, and leukocyte migration, via endocrine, paracrine, and autocrine pathways. Endothelial dysfunction can lead to an imbalance in the production of vasodilatory and vasoconstrictive mediators, resulting in reduced vasodilatory capability of blood vessels, predominantly due to the decrease in nitric oxide (NO) availability [13,14]. One of the main causes of endothelial dysfunction and endothelium activation is the imbalance between reactive oxygen species (ROS) and antioxidants. ROS can influence the onset of endothelial dysfunction via many pathways, including the reduction in NO availability. This can be a consequence of direct reactions between ROS and NO, for example the reaction of the superoxide anion (O_2_•^−^), with NO to form peroxynitrite. Alternatively, ROS can disrupt the synthesis of NO through activation of inflammatory pathways mediated by TNF-α, which can lead to decreased endothelial nitric oxide synthase (eNOS) activity [15]. Importantly, shear stress and oxidative stress are intimately interconnected in vascular pathophysiology. Laminar shear stress enhances antioxidant defenses through Nrf2-mediated upregulation of phase II detoxifying enzymes and antioxidant proteins [16]. In contrast, disturbed blood flow increases ROS production through NADPH oxidase (NOX) activation and endothelial mitochondrial dysfunction while simultaneously impairing antioxidant systems [17]. This mechanistic crosstalk suggests that to accurately model endothelial oxidative stress responses we need to incorporate physiologically relevant flow conditions.

High levels of oxidative stress also play a key role in ischemia-reperfusion injury. During ischemia, ischemic damage to endothelial cells and surrounding tissue is directly proportional to the duration of ischemia. However, the reperfusion injury is caused by the restoration of blood flow. It causes endothelial and tissue damage with a condition of extreme oxidative stress, caused by an influx of neutrophils which produce inflammatory cytokines, free radicals, and vasoactive substances [18,19]. This leads to microvascular damage, which decreases the barrier function of the endothelium and enables the extravasation of blood components and immune cells [18,19,20].

Various endothelial cell types have already been cultured in microfluidic devices, including human umbilical vein endothelial cells (HUVECs), human coronary artery endothelial cells (HCAECs), bovine aortic endothelial cells (BAECs), and immortalized cell lines such as EA.hy926 [5,9,11,12,21]. While primary endothelial cells offer greater physiological relevance, immortalized cell lines such as EA.hy926 provide advantages of consistency, reproducibility, and reduced donor-to-donor variability, making them a valuable tool for standardized assay development and screening applications [22]. The EA.hy926 cell line, derived from fusion of HUVECs with the human lung carcinoma cell line A549, has become a widely used model for endothelial research due to its retention of endothelial characteristics (expression of von Willebrand factor, CD31, VE-cadherin, eNOS) combined with indefinite proliferative capacity [22,23].

Currently available endothelium-on-chip models were developed to study (i) endothelial barrier function and permeability [24]; (ii) leukocyte adhesion and transmigration [9]; (iii) angiogenesis and vascular network formation [25]; and (iv) disease modeling including atherosclerosis, thrombosis, and vascular inflammation [5,11,26]. However, relatively few studies have systematically investigated endothelial responses to oxidative stress under flow conditions in vitro, despite the clear mechanistic rationale for such an approach.

Thus, we aimed to investigate the effects of severe oxidative stress on the endothelium and to develop an endothelium-on-a-chip model for in vitro evaluation of acute oxidative injury. The aim was not to design a new chip architecture, but to develop and document a reproducible, accessible endothelial OOC assay for acute oxidative injury under flow using commercially available components. The OOC model was based on the human endothelial cell line EA.hy926, which was chosen due to its being one of the most studied endothelial cell lines, while offering good repeatability and robustness for method development [22,23,27]. In this study we highlighted the importance of collagen-coating in the preparation of an endothelium-on-a-chip model, developed a method for the determination of cell viability in an OOC, and established two different in vitro models of acute oxidative injury, one based on application of H_2_O_2_ and the other on application of free radical generator AAPH. Together, these experiments provide a methodological framework for the study of acute endothelial injury and screening of potential protective effects of novel pharmacological approaches.

## 2. Results

### 2.1. Optimization of Culture Conditions for Endothelial Growth in Atmospheric CO_2_ Conditions

Due to the use of pressure-based microfluidics, maintaining the system in an atmosphere with 5% CO_2_ was technically challenging. Thus, we optimized culture conditions for cells cultured under ambient CO_2_ conditions. First, we attempted to increase the buffering capacity of DMEM by supplementation with HEPES, which delayed and reduced the increase in medium pH in a concentration-dependent manner. However, the pH increase was outside the acceptable range after 24 h of exposure to atmospheric levels of CO_2_, as it exceeded the pH of 7.4 ± 0.1 even at the highest HEPES concentration (40 mM). The EA.hy926 cell culture also demonstrated the unsuitability of HEPES-supplemented DMEM under these conditions. After cell seeding and 48 h growth in atmospheric conditions, no cell proliferation occurred in HEPES-supplemented DMEM. We therefore selected CO_2_-independent medium, which is specifically formulated for experiments performed in conditions without a regulated CO_2_ atmosphere. This medium maintained a stable pH of 7.3 ± 0.1 for up to 24 h when exposed to atmospheric levels of CO_2_.

### 2.2. Collagen Coating Improves Adhesion and Retention of Endothelial Cells Under Flow

**Figure 1:**
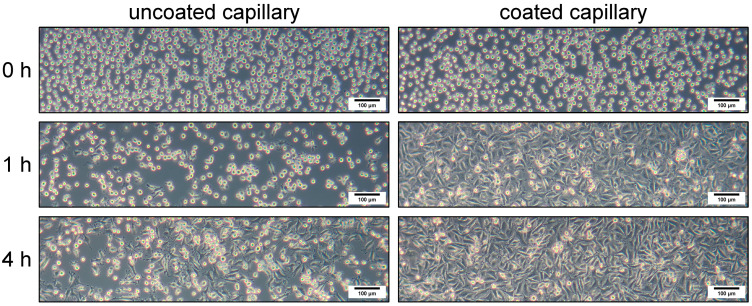
Representative phase-contrast images of EA.hy926 cells seeded in an uncoated capillary (**left**) and a capillary coated with 0.1 mg/mL rat tail collagen type I (**right**) immediately after cell seeding (0 h), 1 h and 4 h after cell seeding and incubation in static conditions. The micrographs were taken at 100× magnification. Cells were seeded into both channels at 1.0 × 10^5^ cells/channel (corresponding to 3.3 × 10^6^ cells/mL, 30 µL per channel and an initial seeding density of 1.46 × 10^5^ cells/cm^2^).

**Figure 2:**
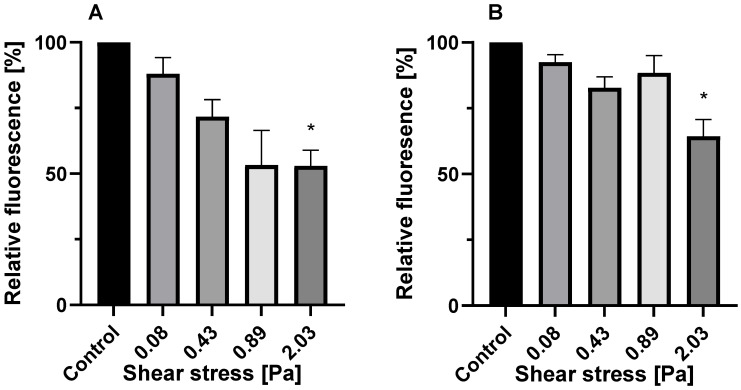
The effect of increasing shear stress on fluorescence measured by the resazurin assay in (**A**) an uncoated capillary and (**B**) capillary coated with 0.1 mg/mL rat tail collagen type I. Control represents cells exposed to culture medium flow corresponding to a shear stress of 0.004 Pa. Data shown as mean ± SEM, (independent biological replicates, *n* = 3 for uncoated capillary, *n* = 4 for coated capillary). One-way repeated measures ANOVA with post-hoc Dunnett test and Geisser-Greenhouse correction; * *p* < 0.05 vs. control.

For cell seeding in the microfluidic chip, the minimal cell suspension concentration to achieve confluence above 90% upon cell attachment was determined at 3.3 × 10^6^ cells/mL. Higher concentrations were avoided to minimize the risk of microchannel obstruction and reduce the number of floating non-adhered cells above the confluent monolayer, which would influence image analysis. Cell adhesion time was markedly shortened in capillaries coated with collagen, as can be seen from the change in cell shape from a round to an attached, spread morphology upon adhesion after 1 h, while cells in the uncoated capillary remained poorly adhered even after 4 h of static incubation (Figure 1). Upon exposure of both capillaries to flow conditions, the capillary without collagen coating showed a greater fluorescence decrease as measured by resazurin assay (Figure 2A) than the collagen-coated capillary (Figure 2B). This could be explained by the wash-out of non-adhered but still viable cells, which were loosely entrapped in the collagen matrix. A shear stress of 2.03 Pa led to a significant decrease in resazurin fluorescence in both uncoated and collagen-coated capillary due to the washout of cells (Figure 2). Thus, our collagen-coated model can tolerate shear stress of up to 0.89 Pa.

Inspection of capillaries under a phase-contrast microscope revealed that cell washout was not homogeneous but rather occurred in patches (Supplementary Figure S1), due to the flow of cell culture medium. This was especially prominent in case of cells grown in uncoated capillaries, which also explains the higher variability observed in this condition, as the washout of larger cell patches was not repeatable (Figure 2A, Supplementary Figure S1). At higher culture medium flow rates, we observed a decrease in cell density, accompanied by changes in morphological properties of cells.

### 2.3. Establishment and Validation of Flow Conditions in the Microfluidic System

**Figure 3:**
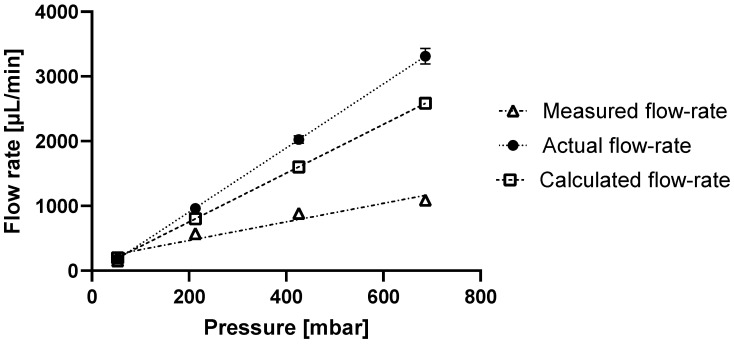
Comparison of the flow-rate, reported by the system (measured flow-rate, Δ), gravimetrically determined flow-rate (actual flow-rate, ●), and flow-rate calculated from applied pressure and system resistance (calculated flow-rate, □) after prolonged cell incubation in the microfluidic system (e.g., >2 days). The discrepancy between the sensor-reported and actual flow rates was attributed to accumulation of cell debris and medium components on the flow sensor.

Longer experiments (e.g., >2 days) revealed a visible difference between the reported flow rate, as measured by the flow meter (Flow unit L), and the actual flow rate in the system, determined gravimetrically (Figure 3). This difference could be attributed to the accumulation of cell debris, washed out cells, and other components of cell culture medium in the capillary of the flow meter, which works on the principle of a microheater with a temperature sensor. If the walls of the measurement capillary become coated with accumulated materials, the heat transfer is modified compared to starting conditions and an incorrect flow rate is reported. Moreover, the maximum flow rate, which can be determined by the Flow unit L, is 1100 µL/min [28], which is below the flow rate required to achieve shear stress above 1 Pascal. Therefore, we opted to use pressure-based flow control, which, although less accurate, enabled more precise flow rates during longer experiments as well as the use of flow rates above 1100 µL/min.

### 2.4. Exposure to Culture Medium Flow Increases Cell Sensitivity to Oxidative Stress

**Figure 4:**
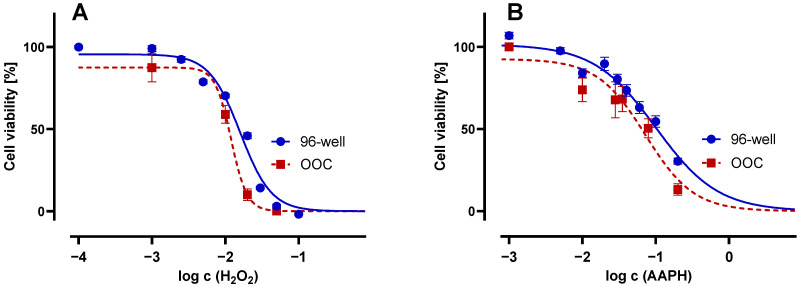
Concentration–response curves for EA.hy926 cell viability after exposure to (**A**) hydrogen peroxide (H_2_O_2_) and (**B**) 2,2′-azobis(2-amidinopropane) dihydrochloride (AAPH) under both static (96-well plate) and flow (organ-on-a-chip, OOC) conditions. Data are shown as mean ± SEM (minimum of 3 independent biological replicates for each condition). Curves were fitted using a four-parameter logistic model (Hill’s equation).

**Figure 5:**
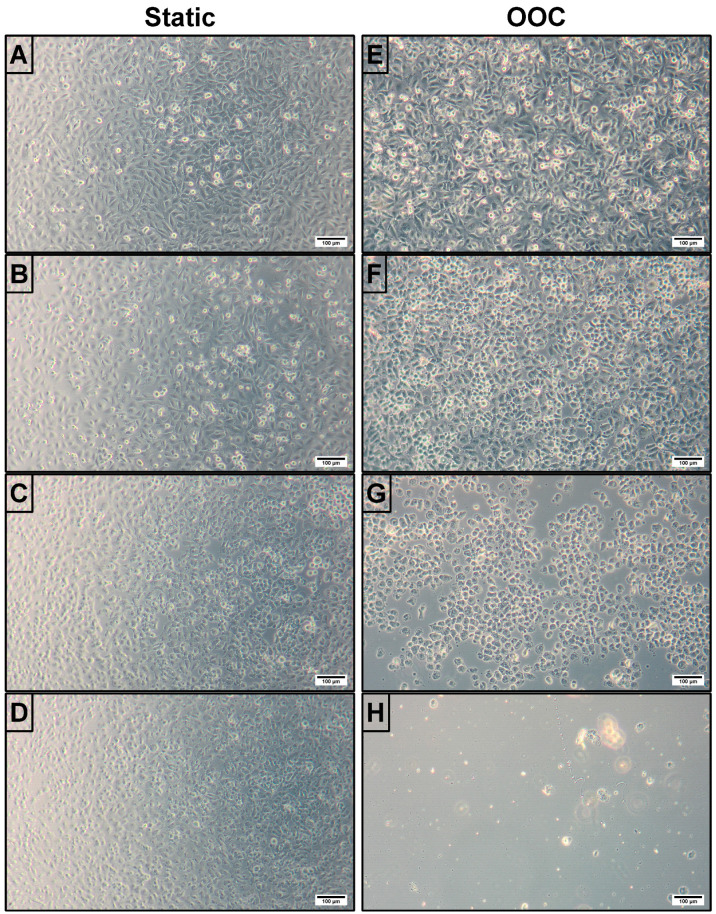
Representative phase-contrast micrographs of EA.hy926 cells after 30 min of exposure to H_2_O_2_ in static (**A**–**D**) and flow (**E**–**H**) conditions. (**A**,**E**)—control; (**B**,**F**)—exposure to 10 mM H_2_O_2_; (**C**,**G**)—exposure to 20 mM H_2_O_2_; (**D**,**H**)—exposure to 50 mM H_2_O_2_. The micrographs were taken at 100× magnification.

**Figure 6:**
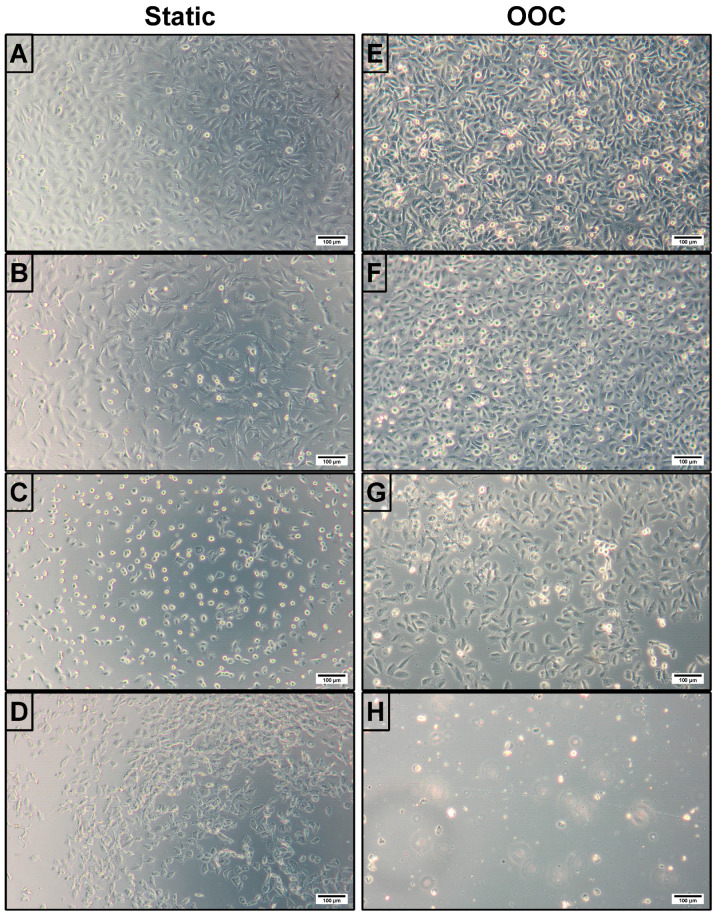
Representative phase-contrast micrographs of EA.hy926 cells after 3 h of exposure to AAPH in static (**A**–**D**) and flow (**E**–**H**) conditions. (**A**,**E**)—control; (**B**,**F**)—exposure to 50 mM AAPH; (**C**)—exposure to 100 mM AAPH; (**G**)—exposure to 79 mM AAPH; (**D**,**H**)—exposure to 200 mM AAPH. The micrographs were taken at 100× magnification.

The exposure of cells to medium flow in the OOC influenced cell viability upon incubation with H_2_O_2_ (Figure 4A) as well as with AAPH (Figure 4B). The decrease in cell viability was more pronounced under flow conditions at 10–20 mM H_2_O_2_ (Figure 4A), possibly due to loss of damaged but still partly viable cells caused by the flow of cell medium (Figure 5). Changes in cell morphology became apparent at 20 mM H_2_O_2_ under static conditions (Figure 5C), while similar changes were already observed at 10 mM H_2_O_2_ under flow conditions (Figure 5F). Moreover, large patches not covered with cells were present in the samples of cells incubated with 10 mM or more of H_2_O_2_ (Figure 5F,G), and no cells remained in the microfluidic channel after incubation with 50 mM of H_2_O_2_ (Figure 5H). Similarly, a difference in cell shape was observed in the case of cells treated with AAPH (Figure 6). Morphological alterations in cell shape were noticeable at a concentration of 50 mM AAPH under flow conditions (Figure 6F), whereas cells remained largely unchanged at the same concentration in static conditions (Figure 6B). Exposure to 100 mM AAPH affected cell morphology in static conditions, with similar changes at 200 mM AAPH (Figure 6C,D). Under flow conditions, such changes in cell morphology were less evident (Figure 6G,H). This was due to the removal of morphologically changed damaged cells by medium flow, leaving patches of the channel not covered with cells in the case of AAPH-treated cells.

Under static conditions, the IC_50_ value for H_2_O_2_ was 16.2 mM (95% CI: 14.9–17.1 mM). In contrast, under flow conditions (0.044 Pa), the IC_50_ was 12.0 mM (95% CI: 10.3–14.3 mM), representing a 26% decrease in apparent IC_50_ under flow conditions. The difference in cell viability between static and flow conditions was most pronounced at intermediate H_2_O_2_ concentrations (10–20 mM).

We used AAPH to complement the H_2_O_2_ model with an exogenous free radical generator, as it produces peroxyl radicals. AAPH-induced concentration-dependent cytotoxicity was observed, with an IC_50_ value of 100.7 mM (95% CI: 89.0–114.6 mM) in static conditions and 72.5 mM (95% CI: 44.2–107.1 mM) under flow conditions (0.044 Pa), representing a 28% decrease in IC_50_ under flow conditions. The enhanced sensitivity to AAPH under flow conditions was consistent across the entire dose-response curve. These findings suggest that medium flow increased the apparent sensitivity of the model to acute oxidative injury.

**Figure 7:**
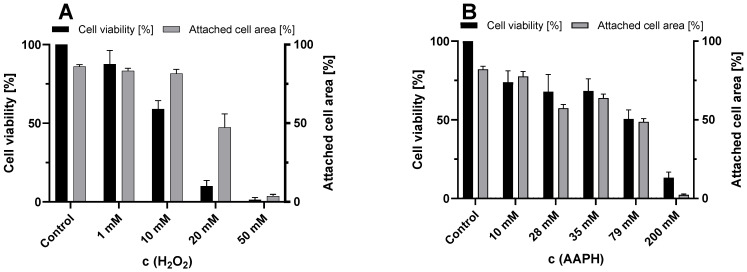
Comparison of resazurin-based cell viability and image analysis-based attached cell area as two readouts of oxidative cell injury under flow conditions after exposure to increasing concentrations of (**A**) H_2_O_2_ and (**B**) AAPH. Control group represents cell viability and attached cell area of cells before treatment with H_2_O_2_ or AAPH. Attached cell area was determined by Ilastik-based image segmentation. Data shown as mean ± SEM.

To quantify the percentage of attached cells and compare it with the data obtained by the resazurin-based cell viability assay, we performed machine-learning-based image analysis using Ilastik software [29]. This analysis was only performed on data obtained from experiments conducted under flow conditions, since the flow of cell medium removes detached cells from the channel. This enabled more reliable quantification of the attached cell area. In static conditions, detached and dead cells remain suspended within the field of view, which interferes with accurate segmentation of surface-attached cells. The area data was not normalized, which resulted in attached-cell area of 86% for H_2_O_2_ control and 82% for AAPH control. This can be explained by detached cells present in the field of view, which cover the underlying attached cells (Figure 5E). For H_2_O_2_-treated cells, the relative decrease in cell viability and change in attached cell area was similar in up to 1 mM exposure (Figure 7A). The difference between cell viability and area of attached cells was greater at intermediate concentrations of H_2_O_2_ (10–20 mM), where cells were still attached (Figure 5F,G) but lost most of their metabolic activity (Figure 7). At these concentrations, the cell shape changes from elongated (Figure 5E) to rounded (Figure 5F,G), with the majority of cells still attached to the OOC surface. Contrary to the image analysis results of cells treated with H_2_O_2_, the image analysis results for cells treated with AAPH showed a strong correlation with the resazurin-based cell viability assay (Figure 7B). This could be explained by the difference in cell morphology between the experiments, as follows: cells treated with AAPH showed minimal change in morphology before detachment (Figure 6E–H), whereas high concentrations of H_2_O_2_ led to morphological changes (Figure 5E–H), which likely affected cell segmentation.

### 2.5. Antioxidant Activity of Quercetin

**Figure 8:**
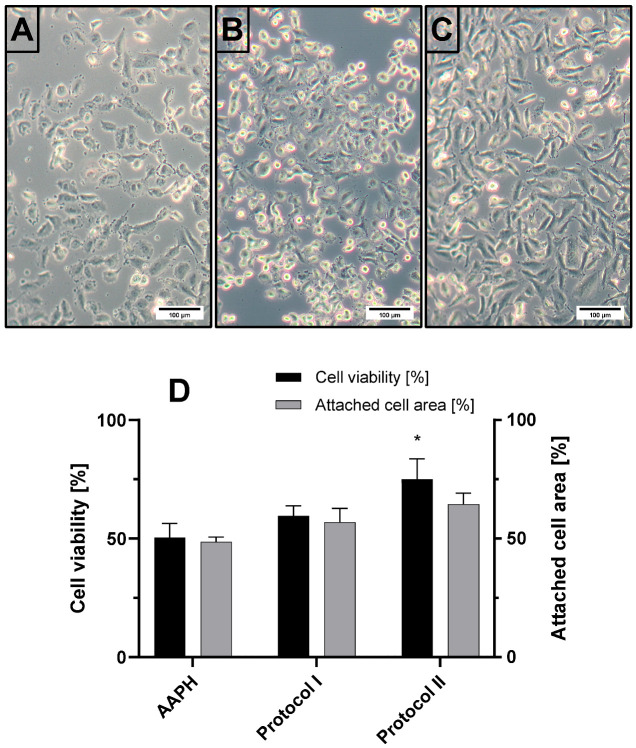
Effect of quercetin on AAPH-induced endothelial injury in the established endothelium-on-a-chip model. The cells were treated for 3 h with (**A**) 79 mM AAPH, (**B**) 1.5 µM quercetin and 79 mM AAPH simultaneously, (**C**) 79 mM AAPH after 30 min preincubation with 1.5 µM quercetin; (**D**) Comparison of resazurin-based cell viability and image-analysis-based attached cell area as two readouts of oxidative cell injury under flow conditions. Protocol I where cells were exposed simultaneously to 1.5 µM quercetin and 79 mM AAPH; and protocol II cells pre-incubated with 1.5 µM quercetin for 1 h, followed by 3 h treatment with 79 mM AAPH. One-way ANOVA with post-hoc Dunnett test and Geisser–Greenhouse correction. Data are shown as mean ± SEM. * *p* < 0.05. The micrographs were taken at 100× magnification.

To evaluate the endothelium-on-a-chip model of AAPH-induced oxidative stress for antioxidant screening, we selected quercetin as a model antioxidant. We used two different incubation protocols, with the cells being exposed to a mixture of quercetin and AAPH in protocol I, and with the cells being pre-treated with quercetin for 30 min, followed by the incubation of cells with the AAPH solution in protocol II. The preincubation with quercetin significantly improved cell viability compared to oxidative stress control (Figure 8D), as it allowed quercetin to enter the endothelial cells. However, exposure to both quercetin and AAPH simultaneously produced only a minimal, non-significant increase in cell viability. Morphological analysis confirmed that quercetin pretreatment preserved endothelial cells and prevented the morphological changes and cell detachment induced by AAPH exposure (Figure 8A–C).

## 3. Discussion

In our study, we developed an endothelium-on-a-chip model, which enables culturing of endothelial cells under conditions of flow. It also supports cell-viability-based as well as image-analysis-based evaluation of acute oxidant-induced endothelial injury. Rather than attempting to reproduce the full complexity of ischemia–reperfusion injury, we aimed to establish a technically accessible endothelial platform that incorporates controlled medium flow and the ability to induce acute oxidative injury. This would allow us to compare endothelial responses under static and flow conditions. From this perspective, the current model should primarily be viewed as a methodological framework for studying acute endothelial injury under conditions that are relevant to reoxygenation and that mimic certain aspects of reperfusion-associated oxidative injury. We developed a human endothelium-on-a-chip platform that integrates physiologically relevant fluid flow with acute oxidative injury modeling. Our principal findings demonstrate the following: (i) EA.hy926 endothelial cells can be cultured and maintained under continuous perfusion at low physiological levels of shear stress (0.08–0.89 Pa); (ii) flow conditions increased cellular sensitivity to oxidative injury induced by both H_2_O_2_ and AAPH, as reflected by 26–28% decrease in IC_50_ values compared to static conditions; and (iii) the model is suitable for screening antioxidant compounds, as demonstrated by quercetin pretreatment.

### 3.1. Establishment of a Medium-Flow-Compatible Endothelial Microfluidic Platform

Collagen coating of microfluidic chip channels was applied because collagen is an important component of the vascular extracellular matrix in the arteries and can promote endothelial cell attachment and spreading on artificial surfaces [30,31]. The use of collagen coating reduced the time required for cells to attach to the surface from over 4 h on an uncoated channel surface to 1 h on the collagen coated channel surface (Figure 1). The extracellular matrix affects integrin activation, leading to the activation of various intracellular signaling pathways [32]. It is therefore crucial for mimicking the conditions in the vasculature in vivo.

Collagen coating also improved cell adhesion in our model when subjected to increasing shear stress. We were able to expose endothelial cells to shear stresses of up to 0.89 Pa without loss of cell viability, while the higher shear stress of 2.03 Pa led to cell detachment and loss of cells from the collagen-coated OOC (Figure 2). Thus, our OOC is suitable to model the shear stress exhibited in vivo in veins and parts of aorta, while further optimization of cell adhesion in the OOC model would be required to mimic the higher shear stress encountered in arterioles [33,34]. Other OOC models recreating an endothelial dysfunction microenvironment report shear stress of 0.3–0.9 Pa in the whole length of a rectangular channel, with a peak of to 1.27 Pa locally on a channel bifurcation [11,12]. This is similar to the capabilities of our model, which can achieve shear stress up to 0.89 Pa. Accordingly, the present system appears suitable for studying endothelial responses under low-to-moderate shear stress. However, it does not yet fully reproduce the higher hemodynamic loads present in all vascular beds.

### 3.2. Establishment of Acute Oxidative Injury Conditions in the Model

Our endothelial model aimed to establish acute oxidant-induced injury similar to the oxidative burden experienced in acute ischemic-reperfusion injury. Thus, we focused on the incubation of endothelial cells with higher concentrations of H_2_O_2_ or AAPH for shorter periods than are generally used in the published studies [27,35,36,37]. We used H_2_O_2_ as a direct oxidant (also mimicking endogenous source of oxidative stress) with pleiotropic cellular effects, while we also used AAPH as a synthetic source of alkoxyl and alkyl peroxyl radicals [38].

Our results in static conditions using shorter incubation periods compared to the published studies fit well with literature data when the differences in exposure time and cell type are taken into account. We obtained an IC_50_ value of 16.2 mM for H_2_O_2_ after 30 min incubation in static conditions, which is significantly higher than the 300 µM reported as a 24 h value of IC_50_ for human umbilical cord vascular endothelial cells (HUVEC) [37]. However, this difference is expected, because substantially higher oxidant concentrations are generally required for short acute exposures than for prolonged incubations. In addition, another study did not determine the IC_50_ value of H_2_O_2_ but showed that incubating the EA.hy926 cells with 0.5 mM H_2_O_2_ for 24 h led to an approximately 40% decrease in cell viability. This establishes the need for much higher concentrations over shorter incubation periods, as well as demonstrates a lower susceptibility of EA.hy926 cells to oxidative stress compared to HUVECs [36].

The change in cell morphology visible in Figure 5C is different from that observed by Seto et. al. [36]. While the cells in their experiments detached from the well surface, visible as white circles with a darker spot under a phase contrast microscope, the cells in our study likely underwent necrotic cell death due to higher concentrations of H_2_O_2_ used. This is evident from their uneven shapes and disconnected cell membranes (Figure 5C,D) [39]. Similar results were obtained with AAPH, where very high concentrations of AAPH were required to decrease cell viability by 50% in 3 h. The obtained IC_50_ value of 100.7 mM after 3 h incubation is significantly higher than the IC_50_ of 5 mM reported for HUVEC cells after 48 h of incubation [40], and 2.5 mM for human microvascular endothelial cells after 24 h incubation [41]. This is in line with our 24 h results for AAPH-induced cytotoxicity, where we obtained the IC_50_ of 10.2 mM. Our result is possibly higher due to the use of hybridoma cells instead of primary HUVEC cells [40,41].

Our image-derived attached cell area analysis provides an additional label-free experimental parameter, which correlates well with resazurin-based cell viability measurements. For AAPH-treated cells, attached cell area and resazurin-based viability showed relatively good agreement. In contrast, for H_2_O_2_-treated cells the correlation was weaker, likely because H_2_O_2_ induced more pronounced cell morphological alterations before detachment. This indicates that attached cell area can provide useful and independent non-invasive measurement of cell status, but should be interpreted with caution when changes in cell morphology are substantial.

### 3.3. Effect of Culture Medium Flow on the Apparent Cell Response to Oxidative Injury

A key finding of the present study was that both H_2_O_2_ and AAPH produced lower apparent IC_50_ values under flow than under static conditions (Figure 4). While it has been shown that flow conditions impact the behavior of the endothelium in vitro [25], we combined shear stress with high levels of oxidative stress to mimic conditions following ischemic-reperfusion injury in vivo with the focus on oxidative stress. We observed a greater decrease in cell viability under flow conditions for cells that were exposed to both sources of oxidative stress. Importantly, this observation should be interpreted cautiously. The stronger response observed under flow conditions does not necessarily reflect only greater intracellular susceptibility to oxidative injury, but also enhanced detachment and washout of injured cells caused by flow. To elaborate further, this could be attributed to cell damage resulting from oxidative stress, due to which cells still preserve some metabolic activity, and thus metabolize resazurin in static conditions. However, the damage to molecules, required for cell adhesion to the surface, leads to cells being carried away with flow of culture medium under flow conditions, as is evident in micrographs (Figure 5 and Figure 6).

From a methodological perspective, this distinction is important, because it suggests that OOC endothelial models reveal an additional dimension of injury related to adhesion failure and cell loss under flow, rather than simply reproducing static oxidative injury results under perfusion. The presented platform may therefore be particularly useful for studying acute endothelial injury in settings where structural retention of the endothelial layer is biologically relevant.

### 3.4. Applicability of the Endothelial OOC Model

Improved cell viability following quercetin pretreatment shows that our model of oxidative stress damage can detect the modulation of oxidant-induced endothelial injury under flow conditions (Figure 8). It can also be used to evaluate antioxidants in conditions, which are more biorelevant than static cultures. In contrast, simultaneous exposure to quercetin and AAPH resulted in only a minimal and non-significant effect. This suggests that pretreatment may be required for intracellular accumulation or preconditioning before protection becomes detectable. In the context of the present study, quercetin should therefore be regarded primarily as a proof-of-applicability intervention rather than as the focus of a detailed pharmacological analysis. Broader validation of the model for antioxidant screening will require testing of additional compounds, concentration ranges, and orthogonal functional endpoints.

Several limitations of the present study should also be acknowledged. Firstly, the model is based on the immortalized EA.hy926 cell line, which is ideal for methodological development due to its high reproducibility and robustness, but does not fully replicate the phenotype or functional diversity of human primary endothelial cells. Secondly, the lower IC_50_ values observed under flow conditions should be interpreted as a combination of decreased metabolic activity, loss of cell adhesion, and washout of cells from the channel. Therefore, our model does not distinguish between true intracellular sensitization and loss of adhesion as a mechanical readout of injury. Also, our system currently models only the acute oxidative injury rather than the entire process of ischemia, reperfusion, leukocyte recruitment, cytokine signaling, and barrier failure that occurs in vivo during acute ischemic events. Moreover, we concluded that the primary endpoints of the current study should be retained cell viability, cell morphology, and attached cell area. Therefore, the model does not yet directly address intracellular ROS generation, nitric oxide (NO) signaling, endothelial barrier permeability, or inflammatory activation. Furthermore, the shear stress range achievable in the present configuration remains limited to relatively low values, while the concentrations of oxidants are significantly higher than those in vivo. Finally, our system is limited by low-throughput, and could benefit from better scalability, which is becoming more common in OOC models [42].

Future work should therefore extend the platform to include primary endothelial cells, broader shear stress ranges, and additional functional readouts, such as barrier integrity, ROS-sensitive signaling, NO-concentration measurements, and characterization of inflammatory markers. Nevertheless, the present model provides a practical basis for the study of acute endothelial oxidative injury under controlled flow conditions and may be useful for the primary screening of novel pharmacological approaches or direct/indirect antioxidants in future investigations of protective interventions.

## 4. Materials and Methods

### 4.1. Chemicals

All materials used were of reagent grade and obtained from commercial sources. Hydrogen peroxide (Hydrogenii peroxidum 3%) was from Lekarne Ljubljana (Ljubljana, Slovenia), quercetin from Biosynth (Bratislava, Slovakia). Dimethylsulfoxide (DMSO) and 2,2′-azobis(2-amidinopropane) dihydrochloride (AAPH) were procured from Sigma Aldrich (St. Louis, MO, USA).

Dulbecco’s phosphate buffered saline (PBS), Dulbecco’s modified Eagle medium—low glucose (DMEM), L-glutamine (200 mM), Penicillin-Streptomycin (10,000 I.U/mL penicillin and 10 mg/mL streptomycin), 4-(2-hydroxyethyl)-1-piperazineethanesulfonic acid (HEPES), and the Type I rat tail collagen were from Sigma-Aldrich (St. Louis, MO, USA), while TrypLE™ Express, fetal bovine serum (FBS), and CO_2_ independent medium were from Gibco (Grand Island, NY, USA). AlamarBlue™ (resazurin) was obtained from Thermo-Fischer scientific (Waltham, MA, USA).

### 4.2. Cell Culture

Human endothelial cell line EA.hy926 (American Type Culture Collection, Rockville, MD, USA), was maintained in standard two-dimensional culture conditions in DMEM supplemented with 10% (*v*/*v*) FBS, 2 mM L-glutamine, 100 IU/mL penicillin and 100 µg/mL streptomycin. The cells were cultured in a humidified atmosphere with 5% CO_2_ at 37 °C. The cells were subcultured twice per week, when they reached 80–90% confluence.

### 4.3. Cell Imaging

The cells were photographed by phase-contrast microscopy (ZEISS Axiocam 208 color, Carl Zeiss Microscopy GmbH, Jena, Germany) after the measurement of cell viability at 40× and 100× magnification (ZEISS Primovert, Carl Zeiss Microscopy GmbH, Jena, Germany). Images were captured with automatic white-balance and brightness adjustment functions integrated in the camera software.

### 4.4. Image Analysis

The phase-contrast images were cropped from the top-left corner to 3500 × 2160 px in Fiji (version 2.16.0/1.54p), to remove the scale bar (custom macro provided as Supplementary File S2), then imported in Ilastik [29] (version 1.4.1), where a pixel classification model was trained using four classes. Classes corresponded to (1) background, defined as regions where no cells or debris were present, (2) attached cells, (3) detached cells, and (4) cell debris. The model was trained on images of control cells acquired before the experiment, where background, attached cells, and detached cells were marked, as well as the last image in a series of experiments, where the background and cell debris were marked (Supplementary Figure S2). Training was performed with all features selected and a brush size of 1, to improve model performance. The training data and images used for analysis are available in Supplementary Materials.

Quantitative image analysis was performed only for experiments conducted under flow conditions, because flow removed detached cells from the channel and allowed more reliable quantification of the attached cell area. Under static conditions, detached and dead cells remained within the field of view and interfered with accurate discrimination between attached and non-attached cells.

The images were then again imported in Fiji for image segmentation and determination of the attached cell area (class 2). An image of .h5 format was imported into Fiji using the Ilastik plugin, and the class corresponding to attached cells was thresholded against all other classes. The total attached cell area and part of the total image area were measured and exported in a .csv file. This workflow was automated using a macro (provided as Supplementary File S3).

### 4.5. Cell Seeding in Microfluidic Chip and Process Optimization

The Be-Flow Standard microfluidic chip (BeOnChip, Zaragoza, Spain) was coated with 0.1 mg/mL collagen, which was prepared from 3.9 mg/mL rat tail collagen type I by dilution with PBS. The collagen solution was kept cool until it was applied to the microfluidic chip. A cool collagen solution (100 µL, 0.1 mg/mL) was applied to the inlet of a capillary. This was followed by incubation (1 h, 37 °C, humid atmosphere) and washing of the capillary with PBS (4 × 100 µL). For OOC experiments cell suspensions were prepared in CO_2_-independent medium supplemented with 10% (*v*/*v*) FBS, 2 mM L-glutamine, 100 IU/mL penicillin and 100 µg/mL streptomycin and seeded into the BeFlow microfluidic chip. Optimization of the cell seeding concentration was performed to reach over 90% confluence upon cell attachment (complete channel coverage). The cell suspensions were seeded in concentrations from 2 × 10^6^ to 10 × 10^6^ cells/mL, and an analysis of micrographs followed after a 1 h incubation in a CO_2_-free incubator (37 °C) to determine cell confluence.

The time required for cell adhesion and necessity of collagen coating was determined on two-channel Be-flow chips, with one capillary coated with collagen while the other was left uncoated as control. The collagen coating was performed following the above protocol. Cells were seeded into both channels at 1.0 × 10^5^ cells/channel (corresponding to 3.3 × 10^6^ cells/mL, 30 µL per channel and an initial seeding density of 1.46 × 10^5^ cells/cm^2^) and incubated in a CO_2_ free incubator (37 °C, humid atmosphere). The cell adhesion time was approximated from cell images taken in 1 h intervals after cell seeding, while the adhesion strength was evaluated by the exposure of cells to flow rates corresponding to the shear stress of 0.08, 0.43, 0.89, and 2.03 Pa for 60 min. Shear stress in the rectangular channel was calculated according to Equation (1):

(1) $$\tau =\frac{6\eta Q}{h^{2}w}$$

where *τ* represents shear stress in Pa, *η* represents dynamic viscosity, *Q* represents flow rate, *h* represents the height of the channel, and *w* represents channel width. The calculation was performed for DMEM with 10% added serum and a viscosity of 0.930 mPas [43]. Following each increase in flow rate, the resazurin cell viability assay was performed, and cells were imaged as described above. Flow rate was determined from system pressure by the use of a shear stress calculator for the system with 270 cm of tubing with an internal diameter of 0.508 mm [44].

### 4.6. Cell Viability Determination

Cell viability in static conditions was determined by a resazurin assay. Following an experiment, the spent culture medium was removed, and the cells were washed once with 100 µL of PBS, followed by an addition of fresh medium containing 10% (*v*/*v*) alamarBlue^TM^ (Waltham, MA, USA). Cell viability was determined after two hours of incubation by determination of fluorescence at 590 nm with excitation at 560 nm using a fluorescent filter in a plate reader (Tecan Spark^®^, Männedorf, Switzerland). Blank was represented as culture medium with 10% (*v*/*v*) alamarBlue^TM^ without cells, while control was represented by cells incubated in culture medium without treatment. Cell viability was calculated as presented in Equation (2):

(2) $$Cell\;viability\;\left(\%\right)=\;\frac{F_{sample}-F_{blank}}{F_{control}-F_{blank}}\times 100$$

where *F*_sample_ represents the sample fluorescence, *F*_blank_ represents the fluorescence background, and *F*_control_ represents the fluorescence of control.

Cell viability in microfluidic experiments was determined and calculated differently than that in static conditions. After incubation under flow conditions, the OOC was disconnected from the microfluidic apparatus, with both capillaries washed with an excess of culture medium containing 10% (*v*/*v*) of alamarBlue^TM^ to remove spent culture medium. Then, all medium in the wells, except that present directly in the capillary, was removed and the OOC was incubated in static conditions (37 °C, 30 min). After 30 min of incubation, 25 µL of the reagent were transferred from the capillary to a 96-well plate and mixed with 25 µL of PBS. Cell viability was determined by measurement of fluorescence at 590 nm with excitation at 560 nm using a fluorescent filter in a plate reader (Tecan Spark^®^, Switzerland). Blank was prepared for each timepoint by mixing 25 µL of 10% (*v*/*v*) solution of alamarBlue^TM^ with 25 µL of PBS. The cell viability was calculated according to Equation (3):

(3) $$Cell\;viability\;\left(\%\right)=\frac{F_{sample}-F_{sample-blank}}{F_{0}-F_{0-blank}}\times 100$$

where *F*_sample_ represents the sample fluorescence, *F*_sample−blank_ represents the blank measured with the sample, *F*_0_ represents the cell viability of the same chip measured before an experiment, and *F*_0−blank_ represents the blank before the experiment.

### 4.7. Microfluidic Cell Culture

All microfluidic experiments were done using a Fluigent pressure-based pump system (Paris, France), which consisted of a pressure generator (FPLG+), two units of FlowEZ^TM^, and two 2-Switches controlled by OxyGEN software (version 2.3.2.0). The flow was measured and regulated by a Flow unit L sensor. Flow rate validation was performed prior to an OOC experiment and after the conclusion of the experiment. The flow validation experiments were performed gravimetrically, setting a specific flow for a defined period, while collecting the efflux into a weighted Eppendorf tube. A recirculation setup, which enabled unidirectional flow of CO_2_ independent medium in the endothelial OOC, was used to perform microfluidic experiments (Schematic and picture available in Supplementary Materials Figure S3).

After cell seeding as described above, the OOC was incubated in static conditions to allow cell adhesion (3 h, 37 °C). The OOC was then connected to the microfluidic recirculation loop with a set flow of 100 µL/min (corresponding to a shear stress of 0.044 Pa) overnight.

### 4.8. Determination of Oxidative Stress in Static Conditions

Endothelial cells EA.hy926 were seeded on the central 60 wells of 96-well plates (1.5 × 10^4^ cells/well) containing 100 µL of complete DMEM and grown for 48 h to reach confluence. Then, the cells were washed with PBS, followed by the addition of H_2_O_2_ or AAPH in complete CO_2_-independent medium.

For H_2_O_2_ experiments, cells were incubated with H_2_O_2_ for 30 min in the following concentrations: 0 (control), 0.1, 1, 2.5, 5, 10, 20, 30, 50, and 100 mM. After the incubation, the H_2_O_2_-containing medium was removed and replaced with fresh CO_2_ independent medium containing 10% (*v*/*v*) alamarBlue^TM^ reagent (Thermo-Fischer Scientific, Waltham, MA, USA); cell viability was determined as described above.

For AAPH experiments, cells were incubated with AAPH for 3 h as well as 24 h in the following concentrations: 0 (control), 0.1, 0.5, 1, 5, 10, 20, 30, 40, 60, 100, and 200 mM. After the incubation, the AAPH-containing medium was removed and replaced with fresh CO_2_ independent medium containing 10% (*v*/*v*) alamarBlue^TM^ reagent, and cell viability was determined as described above.

To control for changes in osmolarity, in a separate set of experiments sodium chloride was added to cell culture medium at concentrations of 5 mM, 25 mM, and 100 mM, corresponding to an increase in osmolarity of 10 mOsm/L, 50 mOsm/L, and 200 mOsm/L.

### 4.9. Determination of Oxidative Stress Under Flow Conditions

Cells were seeded in OOC channels at a concentration of 3.3 × 10^6^ cells/mL, which was the cell seeding concentration used in all oxidative-stress-related OOC experiments. After 1 h of static incubation, we exposed the cells to flow conditions overnight (100 µL/min, corresponding to the shear stress of 0.044 Pa).

Following overnight incubation under flow conditions, the OOC was disconnected from the recirculation loop and the medium inside the capillaries was replaced by a 10% (*v*/*v*) solution of the alamarBlue™ reagent in CO_2_ independent medium (control measurement). The reagent was incubated in static conditions for 30 min, after which 25 µL were removed from each capillary and transferred to a 96-well plate and diluted with 25 µL of PBS. The fluorescence was measured at 590 nm with excitation at 560 nm using a plate reader. Cells were imaged after each time point by phase-contrast microscopy as described above in Section 4.3.

Following the control measurement, the microfluidic OOC was attached back in the recirculation loop with CO_2_ independent medium containing H_2_O_2_ in the lowest concentration (1 mM). The H_2_O_2_ solution was circulated for 30 min (100 µL/min, corresponding to the shear stress of 0.044 Pa), after which the OOC was disconnected, and the cell viability was determined. This protocol was repeated on the same cells in concentrations of 1, 10, 20, and 50 mM of H_2_O_2_. A similar protocol was employed for the incubation of cells with AAPH, with each concentration being recirculated for 3 h instead of 30 min with three subsequent AAPH concentrations evaluated in three groups (10, 50, and 200 mM; 28, 79, and 200 mM or 35, 80, and 200 mM). Concentrations were refined during assay calibration to include low injury, near IC_50_ injury, and near-complete injury.

The applicability of the OOC model was further evaluated using quercetin as a model antioxidant by two different protocols. In protocol I (simultaneous approach), a 1.5 µM solution of quercetin was prepared in a 79 mM solution of AAPH in CO_2_ independent medium (corresponding to a preliminary IC_50_ value under flow conditions). Following the control measurement, this solution was recirculated for 3 h. In protocol II (pre-treatment approach), a separate solution of 1.5 µM quercetin was prepared in CO_2_ independent medium, which was recirculated for 30 min prior to the 3 h of recirculation with 79 mM AAPH, followed by the determination of cell viability.

### 4.10. Statistical Analysis

Data are presented as mean ± SEM. The number of independent biological replicates (n) is indicated in the corresponding figure legends. Statistical analysis was performed using GraphPad Prism version 11.0 (GraphPad Software, San Diego, CA, USA). Differences were considered statistically significant at *p* < 0.05.

For comparison of cell viability across multiple shear-stress conditions within the same experimental series compared to the control group, one-way repeated-measures analysis of variance (ANOVA) followed by Dunnett’s post hoc multiple-comparison test was used.

For concentration–response experiments with H_2_O_2_ and AAPH, we determined IC_50_ values by nonlinear regression using a four-parameter logistic model (Hill equation). Curve fitting was performed separately for static and flow conditions.

For experiments evaluating the protective activity of quercetin on AAPH-induced injury in our OOC model, statistical comparisons between treatment groups were performed using one-way ANOVA followed by Dunnett’s post hoc multiple-comparison test, using the AAPH-treated group as the reference group.

## Data Availability

The data presented in this study are openly available in [Human Endothelium-on-a-Chip: Development of a Microfluidic Model for Cell Viability Assessment under Oxidative Injury—Supplementary Data] [https://doi.org/10.5281/zenodo.19552411].

## Supplementary Materials

The following supporting information can be downloaded at: https://www.mdpi.com/article/10.3390/ijms27115018/s1.

## Abbreviations

The following abbreviations are used in this manuscript:

AAPH	2,2′-azobis(2-amidinopropane) dihydrochloride
H_2_O_2_	Hydrogen peroxide
HUVEC	Human umbilical vein endothelial cells
OOC	Organ-on-a-chip
SEM	Standard error of the mean
ANOVA	One-way repeated-measures analysis of variance
DMEM	Dulbecco’s modified Eagle medium
PBS	Dulbecco’s phosphate buffered saline
FBS	Fetal bovine serum
NO	Nitric oxide
HEPES	2-[4-(2-Hydroxyethyl)piperazin-1-yl]ethane-1-sulfonic acid
HCAECs	Human coronary artery endothelial cells
BAECs	Bovine aortic endothelial cells
NOX	NADPH oxidase
ROS	Reactive oxygen species
IC_50_	Concentration causing 50% reduction in cell viability
