# Supplementary material for: Human Endothelium-on-a-Chip: Development of a Microfluidic Model for Cell Viability Assessment Under Oxidative Injury
Source: Int J Mol Sci. 2026 Jun 2;27(11):5018. doi: 10.3390/ijms27115018 (PMC13256723; doi:10.3390/ijms27115018)
Supplement: Supplementary file 1 [file ijms-27-05018-s001.zip › Supplementary File S1.pdf]

Supplementary data

Human Endothelium-on-a-Chip: Development of a Microfluidic Model for Cell Viability Assessment under Oxidative Injury

Klemen Kirbus [1], Jakob Kolar [1], Črt Krebs [2], Petra Kocbek [1] and Lovro Žiberna [1,2,*]

[1]University of Ljubljana, Faculty of Pharmacy, Aškerčeva cesta 7, 1000 Ljubljana, Slovenia; klemen.kirbus@ffa.uni-lj.si (K.K), jakob.kolar@ffa.uni-lj.si (J.K), petra.kocbek@ffa.uni-lj.si (P.K)

[2]University of Ljubljana, Faculty of Medicine, Korytkova ulica 2, 1000 Ljubljana, Slovenia; crt.krebs@mf.uni-lj.si (Č.K)

*Correspondence: lovro.ziberna@ffa.uni-lj.si (L.Ž);

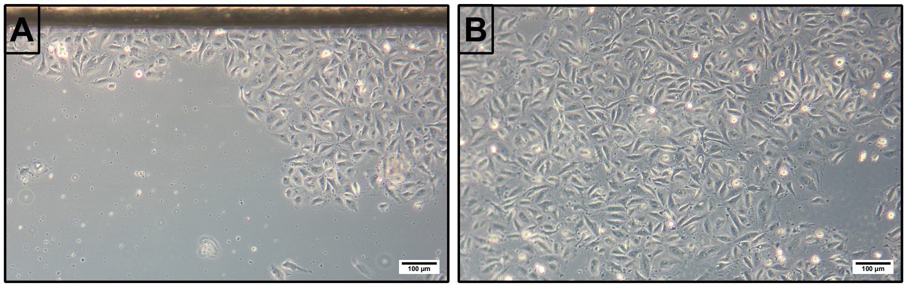

Figure S1. Examples of cell washout in different areas of the same capillary (A) on a larger area and (B) on a smaller area. The micrographs were taken at 100x magnification.

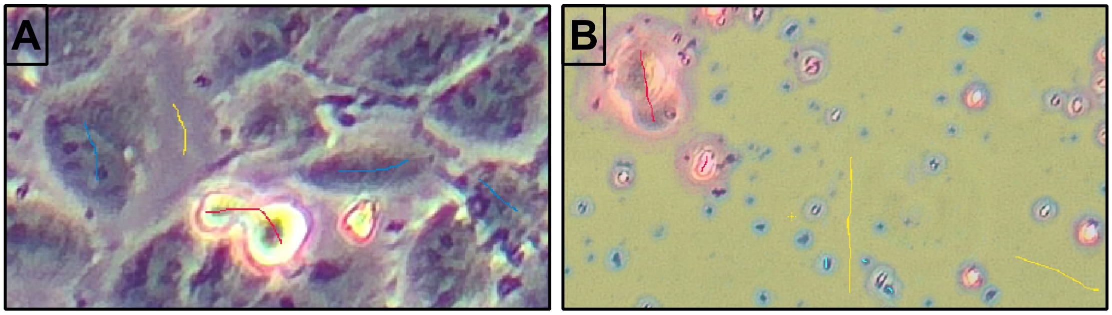

Figure S2. Representative examples of Ilastik training on (A) confluent cells and (B) remains of cells after the final experiment. Stripes represent training data, with four classes, corresponding to background (yellow), attached cells (blue), detached cells (red) and cell debris (cyan). The micrographs were taken at 100x magnification and cropped, to showcase thin lines of training data.

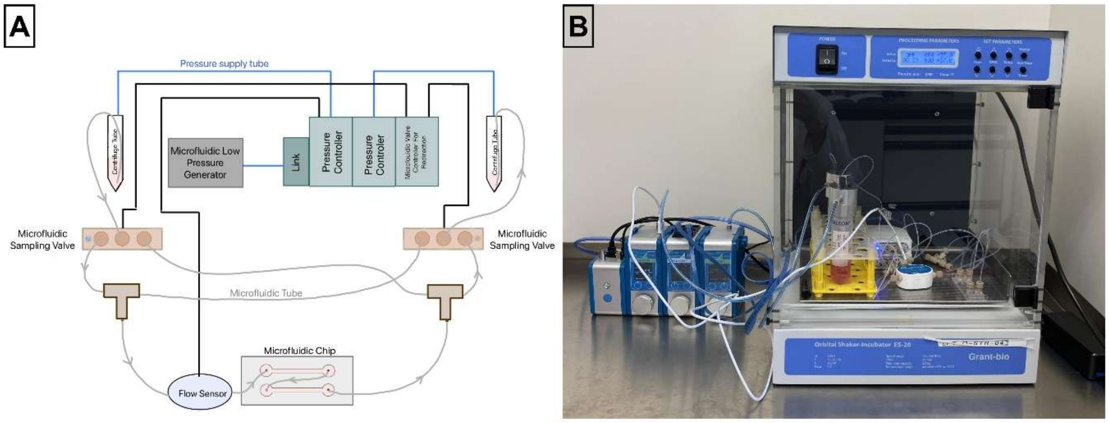

Figure S3. (A) A schematic of the microfluidics apparatus used to perfuse endothelium-on-a-chip and (B) the actual microfluidic apparatus used in the experiments.

The macro files are available as separate files in the Supplementary data.

Fiji macro *1* instructions: open the macro in Fiji, choose input folder when prompted “Choose input folder”, choose output folder when prompted “Choose output folder”.

Fiji macro 2 instructions: open the macro in Fiji, choose input folder when prompted "Choose folder with .h5 files", choose the folder where the .csv file with results will be saved when prompted "Choose output folder for CSV".
